# Reference Intervals in Healthy Adult Ugandan Blood Donors and Their Impact on Conducting International Vaccine Trials

**DOI:** 10.1371/journal.pone.0003919

**Published:** 2008-12-11

**Authors:** Leigh Anne Eller, Michael A. Eller, Benson Ouma, Peter Kataaha, Denis Kyabaggu, Richard Tumusiime, Joseph Wandege, Ronald Sanya, Warren B. Sateren, Fred Wabwire-Mangen, Hannah Kibuuka, Merlin L. Robb, Nelson L. Michael, Mark S. de Souza

**Affiliations:** 1 U.S. Military HIV Research Program, Rockville, Maryland, United States of America; 2 Makerere University Walter Reed Project, Kampala, Uganda; 3 Uganda National Blood Transfusion Service, Kampala, Uganda; 4 Walter Reed Army Institute of Research, Rockville, Maryland, United States of America; 5 Armed Forces Research Institute of Medical Sciences, Bangkok, Thailand; Dalhousie University, Canada

## Abstract

**Background:**

Clinical trials are increasingly being conducted internationally. In order to ensure enrollment of healthy participants and proper safety evaluation of vaccine candidates, established reference intervals for clinical tests are required in the target population.

**Methodology/Principal Findings:**

We report a reference range study conducted in Ugandan adult blood bank donors establishing reference intervals for hematology and clinical chemistry parameters. Several differences were observed when compared to previously established values from the United States, most notably in neutrophils and eosinophils.

**Conclusions/Significance:**

In a recently conducted vaccine trial in Uganda, 31 percent (n = 69) of volunteers screened (n = 223) were excluded due to hematologic abnormalities. If local reference ranges had been employed, 83% of those screened out due to these abnormalities could have been included in the study, drastically reducing workload and cost associated with the screening process. In addition, toxicity tables used in vaccine and drug trial safety evaluations may need adjustment as some clinical reference ranges determined in this study overlap with grade 1 and grade 2 adverse events.

## Introduction

Substantial efforts are underway to develop and test HIV vaccines internationally. The first priority in vaccine development is the evaluation of safety and tolerability in the clinically “normal” adult population. In order to accurately assess what is healthy, reference intervals for standard laboratory tests in the target population are necessary. Use of improper clinical reference ranges may falsely exclude otherwise eligible volunteers from participating in vaccine trials, making the process of trial enrollment and execution more challenging. In addition, clinical reference intervals in a population are necessary in order to accurately assess potential adverse vaccine reactions observed during the course of a clinical trial.

Common practice in Uganda, both in hospitals and research laboratories, is to use the manufacturer's ranges for a given clinical laboratory assay system. Many of these assay systems are procured from Europe or the United States and use reference values based on their populations, which may not be representative of the Ugandan population. Numerous publications describe differences between clinical reference ranges in African populations compared to industrialized countries [1], [2], [3], [4], [5]. A small number of publications address reference values in Uganda [4], [6], but these are limited in scope to hematologic and selected lymphocyte parameters or restricted geographically to rural areas of Uganda.

In November 2004, screening for enrollment in a Phase I HIV vaccine trial to assess the safety and immunogenicity of a multiclade HIV-1 DNA plasmid vaccine (VRC-HIVDNA009-00-VP, Vaccine Research Center, National Institute of Health, Bethesda, Maryland) began at the Makerere University Walter Reed Project (MUWRP) in Kampala, Uganda. [7] The trial enrolled 31 healthy Ugandan volunteers, with inclusion criteria based on clinical reference ranges obtained from US sources. Due to numerous exclusions based on laboratory abnormalities, the screening to enrollment ratio was extremely high (7 volunteers screened for every 1 trial participant). These numbers present enormous logistical, personnel and financial issues for movement of vaccine candidates into phase II and phase III trials, thus more appropriate reference ranges are needed. In addition, population relevant clinical reference ranges would aid clinicians in patient management in Uganda.

This study establishes the reference ranges for hematology and chemistry values in anonymous, healthy, adult Ugandan blood bank donors in the Kampala region and evaluates their potential implications in vaccine trials.

## Materials and Methods

### Blood Bank

The Nakasero Blood Bank, in Kampala, is the central laboratory for the Uganda Blood Transfusion Service (UBTS). The UBTS supplies over 140,000 units of blood per year to Ugandan hospitals. Blood donations are derived from the donation center in Kampala and at multiple donation centers throughout the country. In accordance with Ugandan National blood donation policy and practices, a confidential pre-donation evaluation is conducted by a trained health counselor to determine eligibility to donate blood. Questions include information about blood transfusion history‚ number of sexual partners‚ use of non-sterilized needles‚ prolonged fever or frequent infections‚ history of liver disease or hepatitis‚ and medication history. In addition‚ a general physical examination including general appearance‚ lymphadenopathy‚ weight, height‚ blood pressure‚ and temperature is performed by a health specialist trained by the UBTS. A copper sulfate test is used to ensure adequate hemoglobin levels in blood donors. As a result of the screening process‚ the HIV prevalence in the blood donor population has been reduced from approximately 14 percent in 1987 to 2 percent in 2003. (personal communication, Dr. Peter Kataaha, Director UBTS) These procedures ensured that blood collection occurred in those donors that were generally healthy.

Participation in this study was anonymous as no linkage was present between the blood donor and samples. The only demographic information collected was gender, age and regional collection site. All donors found eligible as a result of the blood bank screening process were asked to participate in this study. Donors willing to participate were required to complete a written donor affidavit form, consenting to donate residual blood for research purposes. The study received ethical approval from the Makerere University Faculty of Medicine Research and Ethics Committee and the Uganda National Council of Science and Technology in Uganda and from the Walter Reed Army Institute of Research Institutional Review Board in the United States.

### Sample collection

After standard blood collection in a polyethylene donation bag, residual blood remaining in the tubing was collected into 3ml EDTA and serum collection vacutainers. The tubing was manually clamped at the bag to prevent backflow of blood and/or anticoagulant from the bag into the tubing. Samples were transported at room temperature in sealed boxes to the College of American Pathologists (CAP) accredited MUWRP laboratory, located in Kampala, Uganda. Only those samples received in the laboratory within 8 hours of phlebotomy were included in the study. All samples were collected between July and September 2005.

### Hematology and Chemistry Testing

Hematology analysis, including a complete blood count and five part differential, was performed on EDTA anticoagulated blood using the Coulter AcT5 diff (Beckman Coulter, Fullerton, California). Serum vacutainers were centrifuged at 800×g and serum was aliquoted for use on Roche Cobas Integra 400 plus analyzer (Roche, Indianapolis, Indiana). Tests were performed on both platforms in strict adherence to manufacturer's instructions.

### Exclusion from reference range data set

All samples were tested for HIV antibody, Hepatitis B surface Antigen, Hepatitis C Antibody, and pregnancy. All test kits were approved by the US Food and Drug Administration (FDA). Samples found to be positive in any of these tests were excluded from the data set.

Initial screening for anti-HIV-1 antibody was conducted using Genetic Systems rLAV ELISA (BioRad Laboratories, Redmond, WA). Reactive samples were repeated in duplicate using the Vironostika HIV-1 Microelisa Systems (Organon Teknika, Durham, North Carolina). Samples repeatedly reactive by both ELISAs were tested using the Genetic Systems HIV-1 Western Blot (BioRad Laboratories, Redmond, WA). Screening for Hepatitis B surface antigen (HBsAg) was performed using the Genetic Systems HBsAg EIA 3.0 (BioRad Laboratories, Redmond, WA). Repeatedly reactive samples were confirmed using the Genetic Systems Confirmatory Assay 3.0 (BioRad Laboratories, Redmond, WA). Screening for anti-Hepatitis C antibody was performed using the Ortho HCV Version 3.0 ELISA Tests System. Repeatedly reactive samples were tested in the Chiron RIBA HCV 3.0 SIA (Chiron Corporation, Emeryville, CA). Serum pregnancy testing was performed on all females using Wampole PreVue hCG cassettes. (Wampole Laboratories, Inc Dist., Princeton, NJ)

### Statistical methods

Ranges were calculated using JMP 5.1 (SAS Institute, Cary, NC, USA). The reference range intervals were calculated as the range between those values at the 2.5% and 97.5% limits for the population after exclusions listed above, stratified by gender, thus providing the reference range encompassing 95% of the population. The non-parametric Wilcoxon test was used to determine any statistically significant differences between laboratory values for men and women.

## Results

### Sample collection results

Of 960 samples collected and received in the laboratory, 8% (78/960) were excluded due to laboratory abnormalities (1% positive for HIV Antibody; 4% positive for Hepatitis B Surface Antigen; 3% positive or indeterminate for Hepatitis C Antibody; 3% of females were pregnant). Another 2% (20/960) were excluded from the analysis due to missing data. Following these exclusions, 862 samples were included for clinical reference range determination. Most analytes had far fewer samples included due to issues with sample volume or test availability (Tables 1 and 2), but the number of subjects tested for each analyte was within the sample size (N = 120) suggested by CLSI.[8] The gender distribution was 20% female and 80% male and the median age was 23 years (male:24, female:20), with a range of 18 to 56. Most volunteers (73%) were below the age of 30.

**Table 1:** Hematology 95% Reference Intervals

	MUWRP Female				MUWRP Male				MUWRP Combined				MGHRange[9]
Test	n	Median	Mean (StDev)	Range	n	Median	Mean (StDev)	Range	n	Median	Mean (StDev)	Range	
* **WBC 10^3^ cells/µL**	140	5.2	5.4 (1.2)	3.2–9.0	520	4.7	4.9 (1.3)	2.8–8.2	660	4.9	5.0 (1.3)	2.8–8.2	4.5–11.0
* **RBC 10^6^ cells/µL**	141	4.4	4.4 (0.5)	3.3–5.3	523	5.0	5.0 (0.6)	3.8–6.1	664	4.9	4.9 (0.6)	3.5–6.1	F: 4–5.2 M:4.5–5.9
* **HGB g/dL**	141	12.8	12.7 (1.6)	9.8–16.2	523	14.5	14.4 (1.4)	11.6–17.1	664	14.1	14.1 (1.6)	10.8–17.1	F:12–16 M:13.5–17.5
* **HCT %**	141	37.8	37.2 (4.6)	28.3–46.8	523	42.6	42.3 (4.1)	33.8–49.5	664	41.7	41.2 (4.7)	31.2–49.5	F:36–46 M:41–53
**MCV fL**	141	86	85.7 (5.7)	74–94.5	523	85	84.8 (6.5)	71–97	664	85	85 (6.3)	71–97	80–100
**MCH**	141	29.5	29.3 (2.2)	24.8–32.7	523	29.2	29.0 (2.6)	23.0–33.8	664	29.3	29.0 (2.5)	23.5–33.7	26.4–34
**MCHC**	141	34.2	34.2 (0.6)	33–35.5	523	34.2	34.1 (0.7)	32.4–35.3	664	34.2	34.1 (0.7)	32.5–35.3	31.0–37.0
**RDW, %**	141	12.7	13.1 (1.5)	11.0–17.3	523	12.8	13.0 (1.4)	10.9–16.8	664	12.8	13.0 (1.4)	11.0–16.8	11.5–14.5
* **PLT 10^3^ cells/µL**	141	259	264 (72)	138–457	523	209	215 (61)	106–362	664	218.5	225 (67)	109–384	150–300
* **MPV**	141	7.9	8.0 (0.8)	6.6–9.9	522	8.1	8.2 (0.9)	6.8–10.2	663	8.1	8.1 (0.9)	6.7–10.1	
* **NE%**	140	42.4	42.1 (9.4)	23.8–61.1	520	38.3	39.2 (9.6)	21.7–59.2	660	38.9	39.9 (9.6)	22.2–59.3	40–70
* **LY%**	140	43.7	43.9 (8.7)	26.5–62	520	44.5	44.2 (8.9)	26.6–61.3	660	44.4	44.1 (8.8)	26.7–61.2	22–44
* **MO%**	140	7.5	7.5 (1.8)	4.4–11.4	520	7.7	8.0 (2.0)	4.7–12.8	660	7.7	7.9 (2.0)	4.7–12.7	4–11
* **EO%**	140	3.2	5.8 (5.9)	0.75–22.6	520	5.6	7.9 (6.8)	1.1–25	660	5.0	7.5 (6.7)	1.0–25.0	0–8
* **BA%**	140	0.6	0.6 (0.2)	0.3–1.3	518	0.6	0.7 (0.3)	0.3–1.5	657	0.6	0.7 (0.3)	0.3–1.4	0–3
* **NE # 10^3^ cells/µL**	140	2.2	2.3 (0.8)	1.1–4.4	520	1.82	1.9 (0.8)	0.9–3.8	660	1.9	2.0 (0.8)	0.9–3.9	
* **LY #, 10^3^ cells/µL**	140	2.3	2.3 (0.6)	1.3–3.7	519	2.1	2.1 (0.6)	1.2–3.6	659	2.1	2.2 (0.6)	1.2–3.7	
**MO # 10^3^ cells/µL**	140	0.4	0.4 (0.1)	0.2–0.6	519	0.4	0.4 (0.1)	0.2–0.7	659	0.4	0.4 (0.1)	0.2–0.7	
* **EO # 10^3^ cells/µL**	140	0.2	0.3 (0.4)	0.04–1.4	519	0.3	0.4 (0.4)	0.04–1.7	659	0.2	0.4 (0.4)	0.04–1.6	
**BA # 10^3^ cells/µL**	140	0.03	0.03 (0.01)	0.01–0.07	517	0.03	0.03 (0.02)	0.01–0.08	656	0.03	0.03 (0.02)	0.01–0.08	

* indicates parameters with statistically significant gender differences.

**Table 2:** 95% Chemistry Reference Intervals

	MUWRP Female				MUWRP Male				MUWRP Combined				MGH Range[9]
Test	n	Median	Mean (StDev)	Range	n	Median	Mean (StDev)	Range	n	Median	Mean (StDev)	Range	
* **Albumin (g /dL)**	149	4.32	4.4 (0.3)	3.7–5.2	217	4.68	4.7 (0.4)	3.9–5.4	366	4.5	4.5 (0.4)	3.8–5.3	3.3–5.0
**Alkaline Phosphatase (U/L)**	152	82	85 (28)	47–160	281	76	81 (26)	42–159	433	79.5	83 (27)	44–151	30–120
* **Alanine Aminotransferase (U/L)**	172	11.5	13.2 (8.2)	5.3–39.9	677	16.2	18.7 (9.5)	7.2–43.3	849	15	17.6 (9.5)	6.6–42.8	0–35
**Amylase (U/L)**	152	88.7	93.7 (33.0)	44–177	281	95.2	98 (33.5)	46–175.	433	93.7	96.6 (33.3)	45.6–173.6	60–180
* **Aspartate Aminotransferase (U/L)**	170	15.8	16.9 (4.6)	11.4–28.8	675	20.6	21.6 (6.0)	13.2–35.9	845	19.7	20.6 (6.0)	12.3–34.8	0–35
* **Bilirubin Dir (mg/dL)**	157	0.09	0.1 (0.08)	0.0–0.4	322	0.2	0.2 (0.1)	0.1–0.5	479	0.12	0.16 (0.1)	0.02–0.4	0.1–0.3
* **Bilirubin Tot (mg /dL)**	170	0.6	0.7 (0.4)	0.3–1.9	679	0.8	1.0 (0.6)	0.4–2.6	849	0.8	0.9 (0.5)	0.4–2.5	0.3–1.0
**Calcium (mg /dL)**	171	9.1	9.0 (0.8)	5.3–10.1	680	9.1	9.1 (1.1)	7.5–10.1	851	9.1	9.1 (1.0)	7.1–10.1	9–10.5
**Chloride (mmol /L)**	171	101.0	100.9 (1.9)	97.4 104.5	676	100.7	100.6 (2.3)	96–104.6	847	100.8	100.7 (2.2)	96.0–104.5	98–106
**Cholesterol (mg dL)**	148	151	156 (34)	100–230	214	150	154 (40)	90–235	362	150	155 (38)	91–233	<200
* **Creatine Kinase (U/L)**	170	91.0	110 (80)	46–269	673	162	191(115)	65–497	843	141	175 (114)	55–476	F: 40–150 M:60–400
**Carbon Dioxide (mmol /L)**	141	24.3	23.9 (3.8)	17.0–30.5	532	24.2	23.6 (4.3)	14.3–31.1	673	24.3	23.7 (4.2)	14.5–31.1	21–30
* **Creatinine (mg /dL)**	171	0.7	0.7 (0.1)	0.5–0.9	680	0.82	0.8 (0.1)	0.6–1.2	851	0.8	0.8 (0.2)	0.5–1.2	<1.5
* **Gamma-Glutamyl Transferase (U/L)**	159	16.6	18.4 (9.1)	8.0–41.3	328	21.6	26.2 (16.7)	8.7–70.7	487	19.4	23.7 (15.1)	8.5–68.5	1–94
* **Lactate Dehydrogenase (U/L)**	164	177	187 (42)	130–298	639	192	204(5)	131–340	803	189	201 (51)	131–332	100–190
* **Lipase (U/L)**	149	27.5	29.3 (10.5)	14.0–59.6	214	28.8	32.9 (13.8)	14.7–71.1	363	28.4	31.5 (12.7)	14.1–67.8	0–160
**Magnesium (mmol /L)**	153	0.81	0.8 (0.1)	0.4–1.0	288	0.8	0.8 (0.1)	0.4–1.0	441	0.8	0.8 (0.1)	0.4–1.0	0.8–1.2
**Phosphate (mg /dL)**	172	3.7	3.8 (0.8)	2.5–5.6	680	3.4	3.6 (1.1)	2.3–6.3	852	3.5	3.7 (1.1)	2.3–6.3	3–4.5
**Potassium (mmol /L)**	172	3.9	4.0 (0.4)	3.4–4.8	677	4.0	4.0 (0.4)	3.4–4.8	849	4.0	4.0 (0.4)	3.4–4.8	3.5–5
* **Protein (g /dL)**	159	7.63	7.7 (0.6)	6.8–9.0	328	7.5	7.6 (0.6)	6.5–8.9	487	7.6	7.6 (0.6)	6.6–8.9	5.5–8.0
* **Sodium (mmol /L)**	172	137.7	138 (2.6)	135–146	677	138.7	139 (3.3)	136–148	849	138.6	139 (3.2)	135.3–147.6	136–145
* **Triglyceride (mg /dL)**	159	74.4	88.3 (50.6)	34–206	321	91	110.4 (65)	39–299	480	84.9	103 (61)	39–281	<160
* **Blood Urea Nitrogen(mg /dL)**	172	7.5	8.0 (2.3)	4.4–14.1	676	8.7	9.1 (2.8)	4.7–15.8	848	8.5	8.9 (2.7)	4.6–15.5	10–20
* **Uric Acid (mg /dL)**	162	4.1	4.3 (1.0)	3.0–6.8	567	5.4	5.5 (1.1)	3.5–8.0	729	5.2	5.2 (1.1)	3.3–7.8	F:1.5–6 M:2.5–8

* indicates parameters with statistically significant gender differences.

### Hematology and Chemistry Reference Ranges

Hematologic reference ranges, mean and median values are presented in Table 1. Statistically significant (p<0.05) gender differences were seen in most parameters, with the exception of the red blood cell indices (MCV, MCH, MCHC), absolute monocyte count and absolute basophil count, but these differences were not clinically relevant. In comparing these values with those obtained from the Massachusetts General Hospital (MGH) in the United States [9], there were several noticeable differences. The lower limit of the neutrophil percentage range was considerably decreased in the Ugandan population (22%) compared with the MGH population (40%), while the upper limit of eosinophil percentages was drastically increased (25% in Uganda vs. 8% at MGH). The lower range of the red blood cell parameters (RBC, Hemoglobin, Hematocrit) was also decreased in the Ugandan population.

Chemistry reference intervals were established for 24 analytes including electrolytes, liver function tests, renal function tests, lipid profile, cardiac enzymes and others. Mean and median values along with the 2.5–97.5% ranges are presented in Table 2. There were statistically significant (p < 0.05) differences between men and women for fifteen laboratory parameters. Most tests were in agreement with reference intervals published in the US.[9] Differences in cholesterol and triglyceride values are likely due to the fact that samples in our study were not collected from fasting individuals. Two enzymes, Creatine Kinase (CK) and Lactose Dehydrogenase (LDH) had upper ranges that were substantially higher than the published MGH ranges(See Table 2)[9]. In contrast, the upper limit of the Lipase range was much lower in the Ugandan population (68 U/L) compared to the MGH upper range (160 U/L) although it was within the range given by the instrument manufacturer. (13–60 U/L).[10]

## Discussion

Reference ranges were established for hematology and chemistry parameters using samples derived from anonymous blood bank donors. Although the number of females was disproportionate to males (20% vs. 80%) in this study, the number of females included in reference range determination for each analyte was well within the guidelines of CLSI of 120 subjects[8]. The lower proportion of females is likely due to less frequent participation in blood drives and other medical research due to cultural issues. The low prevalence of Hepatitis B surface antigen, Hepatitis C Antibody and HIV antibody in this group demonstrates the success of the blood bank's screening questionnaire as a method for eliminating donors with pathological conditions or acute illnesses that may have an impact on blood safety. Although a detailed medical history outlined by the CLSI guideline[8], including history of tobacco use, diet, alcohol consumption, fasting status, exercise history, genetic or environmental factors, occupation and socio-economic status could not be obtained and utilized in this study, the rigorous screening process employed by the blood bank presumably resulted in collection from overall healthy adults. Furthermore, the blood bank population is likely to be similar to one that would participate in vaccine trials, making these reference ranges directly applicable to that population.

Hematology ranges derived in this study were comparable to published values in Uganda using identical methodology[4], despite the fact that the previous study focused on rural areas and the health status of the volunteers was largely unknown. Red blood cell parameter and platelet findings were consistent with other published African values[2], [4], [5], with Ugandans having lower levels than subjects from industrialized nations. As has been previously shown in other African countries[11], [12], [13], our analysis revealed drastically higher eosinophil counts than published values derived from the United Sates[9] and is likely a result of the high prevalence of parasitic infestations in Africa[4]. The observed neutropenia in this study has also been previously documented[1], [11], [12], [14], 15, although the cause is still unknown. In contrast with other African studies[2], [4], [16], our data showed significant gender differences between white blood cells and most differential parameters.

Clinical chemistry reference values have not been previously published in Uganda. In contrast with hematologic ranges, chemistry ranges were similar to those published in the United States with a few exceptions. CK and LDH values were substantially higher than those published for the US. Exercise is known to elevate levels of these enzymes[17] and perhaps the topography of Kampala combined with the socio-economic status of the donors compared to the US and Europe may lead to more daily physical exercise in the Ugandan group. Additionally, racial differences in creatine kinase levels have been documented and may be contributing to this disparity.[18]

Calculation of region specific reference ranges is not only important for improving quality of health care, but also for implementation of vaccine trials. The impact of utilization of local reference ranges may be significant in reducing screening to enrollment ratios in clinical trials. Our program recently screened 223 volunteers for a phase I vaccine trial in Kampala, Uganda in 2004–2005. As local reference ranges had not been established, US based reference ranges were used for inclusion/exclusion criteria. These inclusion/exclusion criteria included: 1) White blood cell count: 3,300–12,000 cells/µl; 2) Hemoglobin: >11g/L (females) and >12.5 g/L (males); 3) Neutrophils: 32–66% or >1,500 cells/µl 4)Lymphocytes: 28–61% or >800 cells/µl; 5) Eosinophils: 0–8% or ≤400 cells/µl; 6)Platelet count: 125,000–550,000 cells/µl. A total of 69 volunteers were excluded due to apparent hematology abnormalities. However if locally derived reference intervals had been utilized, 57 of these volunteers (83%) could have been included (Table 3). Additionally, the time period for trial enrollment could have theoretically been reduced from 4 months to approximately 3 months. A reduction in the number of volunteers screened would impact dramatically both daily workload and cost. For example, the personnel cost (clinic and laboratory) for conducting screening visits for the additional 57 volunteers in this vaccine study was approximately $17,000 (US). The extra clinical supplies and lab tests associated with the additional screening cost approximately $10,300 (US). In personnel and lab supply costs alone these additional volunteers increased the cost of the study by $27,000 (US).

**Table 3:** Vaccine Trial Exclusions

	# excluded	# excluded if updated ranges used
**Neutropenia**	24	3
**Eosinophilia**	27	4
**Neutropenia+Eosinophilia**	9	2
**Low Hemoglobin**	4	0
**Thrombocytopenia**	2	1
**Leucopenia**	3	2
**Totals**	**69**	**12**

Equally important, the toxicity tables used for grading adverse events may need re-evaluation based on clinical reference ranges from the developing world (Table 4). In particular, the NIH Division of AIDS (DAIDS) table for grading the severity of adult and pediatric adverse events (http://www3.niaid.nih.gov/research/resources/DMIDClinRsrch/toxtables.htm) used in many clinical trials may not reflect locally established country specific reference ranges. For example, the range for absolute neutrophils established in this study has a lower limit of 0.9×10ˆ3 cells/ul, although this value would qualify as a grade 2 adverse event. Similarly, ranges for hemoglobin, platelets and CO_2_ coincide with a grade 1 adverse event.

**Table 4:** DAIDS Toxicity Table Overlap, the Grade refers to the Adverse Experience

	MUWRP Range	Grade 1 Mild	Grade 2 Moderate	Grade 3 Severe	Grade 4 Potentially Life Threatening
**Absolute Neutrophils (10^3^ cells/µL)**	**0.9–3.9**	1.0–1.3	**0.75–0.99**	0.5–0.749	<0.5
**Hemoglobin (g/dL)**	**10.8–17.1**	**10–10.9**	9–9.9	7–8.9	<7
**Platelets (10^3^ cells/µL)**	**109.0–384.4**	**100–124**	50–99	25–49	<25
**CO2 (mmol/L)**	**14.5–31.1**	**16-<LLN**	11–15.9	8–10.9	<8

Ideally, a reference range study should be conducted as an independent investigation, with carefully designed questionnaires, exclusion/inclusion criteria and a comprehensive sampling plan, according to CLSI guidelines[8]. These studies are time consuming, logistically difficult and quite expensive. Most published reference range studies in Africa [2], [3], [4], [5], [6] have used existing studies/protocols (cohort development, HIV studies, etc) in order to gather data. Some studies have had strict criteria for sample inclusion [2] while the two studies in Uganda required only a negative HIV test [4], [6]. Our study used a similar approach as the previous African studies, although a brief physical exam as described earlier went one step further towards ensuring participation of generally healthy donors. Several limitations are apparent in the design of our study, mostly importantly the lack of detailed information to rule out subclinical conditions. We conducted this study within the framework of the bloodbank's existing procedures, without additional staff or infrastructure and without resources spent on volunteer recruitment. In order to minimize interference in daily activities, very limited extraneous information was collected. A second limitation of this study is the calculation of ranges based on a very specific population. Although the blood bank population can be described as a self-selected population, this is in fact, similar to a population that might participate in clinical trials. In a developing country, where resources are limited, we believe that our study methods were adequate to determine reference ranges in a setting where this type of information is limited.

Despite the stated limitations of the study, we assert that the ranges generated in this study would be suitable for use in a more generalized setting, especially in the absence of other relevant data. It is possible that these ranges could be extrapolated to other regions in East Africa or other regions of Africa, of similar altitudes and environments, however small bridging studies according to CLSI guidelines[8] should be performed in order to validate these ranges in a new population. Our study confirms previous findings that regional differences exist for clinical reference intervals. These intervals are critical for successful evaluation of vaccines and drugs internationally and also for advancing basic health care services in the developing world.
